# Multimodal cognitive and behavioral interventions for patients with MCI: a systematic review and meta-analysis on cognition and mood

**DOI:** 10.3389/fnagi.2024.1390699

**Published:** 2024-04-30

**Authors:** Gelan Ying, Ambar Perez-Lao, Michael Marsiske, Shellie-Anne Levy, Glenn E. Smith

**Affiliations:** Department of Clinical and Health Psychology, University of Florida, Gainesville, FL, United States

**Keywords:** mild cognitive impairment, multimodal interventions, systematic review and meta-analysis, cognitive interventions, behavioral interventions, dementia

## Abstract

**Background:**

Research has increasingly suggested a benefit to combining multiple cognitive or behavioral strategies in a single treatment program for cognitively impaired older adults. Therefore, this systematic review and meta-analysis aimed to summarize results on the effects of multimodal cognitive and behavioral interventions versus control conditions on changes in cognition and mood in patients with mild cognitive impairment (pwMCI).

**Methods:**

The review followed a general PRISMA guideline for systematic literature review with a format consisting of participants, interventions, comparators, and outcomes (PICO). Multilevel meta-analyses of aggregated efficacy were performed to assess the pooled effect sizes for cognitive and mood outcomes. Risk-of-bias, heterogeneity across studies, and publication bias were assessed for each outcome.

**Results:**

After primary and reference searches, 18 studies with low or some concerns of risk of bias were included. Low heterogeneity was found for mood and cognition. Funnel plots did not indicate publication bias. All the studies assessed changes in cognition (*n* = 1,555) while seven studies with mood outcomes (*n* = 343) were included. Multilevel meta-analyses demonstrated moderate effect (Hedge’s *g* = 0.44, 95% CI = [0.21–0.67]) in cognitive outcomes and large effect in mood (*g* = 0.65, 95% CI = [0.37–0.93]). Subdomain analyses found low-moderate effects in global cognition, verbal and non-verbal memory, executive function, visuospatial abilities, and semantic fluency (0.20 < *g* < 0.50).

**Conclusion:**

These findings showed comparable to larger effects of multimodal cognitive and behavioral interventions on cognition than pharmacological treatment. Future studies should focus on the longitudinal effects of multimodal interventions in delaying dementia.

**Systematic review registration**: PROSEPRO, CRD42022349297.

## 1 Introduction

### 1.1 Behavioral interventions for mild cognitive impairment

Mild cognitive impairment (MCI) is a prodromal stage of Alzheimer’s disease (AD) and other types of dementia. In patients with MCI (pwMCI), circumscribed cognitive abilities are commonly below age expectation despite generally intact daily functioning (Petersen, 2004; Smith and Bondi, 2013). However, while pwMCI remain independent in primary daily activities, they may encounter difficulties performing complex functional activities (e.g., managing finances, medications, or shopping) and request increased caregiver attention (Albert et al., 2011). MCI is associated with an approximate 12% annual conversion rate to dementia while the comparable normal control group rate is only 1–2% (Petersen et al., 1997, 2001; Shah et al., 2000). In longer-term follow-up studies approximately 80% of pwMCI converted to dementia within six years (Petersen et al., 1999).

While neurodegenerative forms of dementia are irreversible, non-pharmacological interventions (i.e., behavioral interventions such as physical exercise, note taking, social engagement, and computerized cognitive training) administered at an early stage (e.g., MCI) can preserve functional independence, slow cognitive decline, and thereby delay the onset of dementia (Gauthier, 2005; Levy et al., 2022). A review by Chandler et al. (2016) revealed the benefits of behavioral interventions in improving mood (*k* = 26, Cohen’s *d* = 0.16, 95% CI = [0.03–0.28]), functional ability (*k* = 31, *d* = 0.23, 95%CI = [0.16–0.47]), and metacognition (*k* = 26, *d* = 0.30, 95%CI = [0.15–0.58]) in pwMCI (Chandler et al., 2016). Since that review, numerous additional multicomponent interventions have been reported in pwMCI or other at-risk groups. Large multicomponent behavioral interventions such as Vivifrail, which consisted of physical resistance, balance, flexibility, and gait-retraining exercises for three months, have shown significant improvements in functional capacity, cognitive function, and depression (Casas-Herrero et al., 2022). Alternative interventions including lifestyle training might also play an essential role in mood and functional improvement (Gale et al., 2019; Yu et al., 2019).

These observed benefits lead to hypotheses that repeated cross-domain training might stimulate “compensatory scaffolding” and neuroplastic reorganization (Sherman et al., 2017). In other words, the combination of several approaches in a multicomponent treatment program interventions targeting multiple domains may exhibit additive efficacy. In one systematic review only multicomponent (*k* = 16, Hedges’ *g* = 0.40, 95%CI = [0.16, 0.63]) and multidomain-focused cognitive training (*k* = 13, *g* = 0.23, 95% CI = [0.108, 0.352]) yielded statistically significant improvement in cognitive outcomes post-intervention in pwMCI when compared to MCI controls (Sherman et al., 2017). Thus, combining multiple interventions has been increasingly emphasized as a tool to facilitate functional retention. Previous systematic reviews and meta-analyses have reported benefits in combining physical exercises with cognitively challenging activities in both clinical and non-clinical older adults (Zhu et al., 2016; Gheysen et al., 2018; Gavelin et al., 2021). In one meta-analysis, combined cognitive-physical interventions showed small-to-medium positive effects (*k* = 10, standardized mean difference (SMD) = 0.32, 95%CI = [0.17–0.47]) on global cognitive function and moderate-to-large effects (*k* = 4, SMD = 0.65, 95%CI = [0.09-1.21]) on activities of daily living (ADL) in MCI or dementia patients (Karssemeijer et al., 2017). In contrast, despite the significant benefits evidenced in most studies, a recent systematic review found no difference between combined cognitive-physical training and interventions with isolated elements in executive function, processing speed, attention, mood, and cardiorespiratory fitness (Yang et al., 2020). However, the review focused primarily on cognitive outcomes, which might not reflect the overarching efficacy of multimodal interventions across domains (e.g., quality of life and independent daily functioning).

### 1.2 Gaps in current systematic literature review and meta-analysis

A few limitations were identified in existing systematic literature reviews and meta-analyses. First of all, while the effects of combined interventions have been extensively studied in the past decade (see Supplementary material A), research has focused predominantly on comparative effectiveness analysis (Amofa et al., 2021; Levy et al., 2022), a tool commonly used to explore the additive effect of a specific arm instead of changes an overall program has exerted. For example, Imaoka et al. (2019) used comparative effective analysis to investigate the additive effect of soy peptide as a supplement to memory exercise in pwMCI but did not study the overall efficacy of both when compared to an untreated control group. Secondly, some studies and reviews have mixed samples of pwMCI with healthy older adults or early dementia patients (Li et al., 2011; Straubmeier et al., 2017; Bruderer-Hofstetter et al., 2018; Stephen et al., 2019; Santos Lopes da Silva et al., 2023) due to the small amount of available literature (Gheysen et al., 2018, *k* = 9; Han et al., 2022, *k* = 3; Karssemeijer et al., 2017, *k* = 5). Nevertheless, primary preventions in cognitively healthy older adults can serve distinctive roles from interventions for those with known risk of decline (i.e., secondary preventions). Secondary preventions usually incorporate compensation training and adjustment-related treatments to slow or prevent further decline (Smith, 2016). On the other hand, tertiary preventions for those with dementia diagnoses rely heavily on participants’ capacity to grasp the ideas, which might include differential strategies and evaluation systems from interventions designed for pwMCI. Thus, an essential question regarding the effectiveness of multimodal intervention as a secondary prevention in pwMCI remains unclear. Thirdly, there is a lack of consensus on targeted outcomes. Some studies focused primarily on mobility (Kiper et al., 2022; Mai Ba and Kim, 2022) while others focused on cognition (Yan et al., 2022). Lastly, while one meta-analysis (Meng et al., 2022) has synthesized clinical trials combining cognitive intervention and physical exercise on multiple cognitive domains in pwMCI, this meta-analysis excluded behavioral interventions other than physical exercise and included single intervention comparisons to study the additive effects instead of the overall impact of multimodal interventions. Furthermore, this study also suffered from a limited number of reports (*k* = 8) of randomized control trials (RCTs).

In addition, the definition of “multimodal” varied across studies and was often mixed with terms including “multicomponent” or “multifaceted.” For example, a combination of different physical exercises (Lau et al., 2015; Trautwein et al., 2020; Barisch-Fritz et al., 2022) or cognitive training targeting multiple domains (Tsolaki et al., 2011; Olchik et al., 2013) were treated as multimodal in several studies. While these interventions have included multiple strategies, the target was often limited to one area of concern instead of a comprehensive approach that can target multiple interrelated areas of concern simultaneously. Studies have also used the term “multimodal” to describe treatments conducted in different settings (e.g., home vs. clinic) or through different delivery methods (e.g., computer vs. paper). To establish an operational definition and delineate the targeted treatment types for this review, multimodal interventions generally refer to combining several training approaches that target different outcome domains in a treatment program (Giusti et al., 2017).

In summary, we believe that examining truly multimodal interventions that focus on or at least partition pwMCI for separate analysis might assist future explorations of comprehensive and efficient intervention programs for persons at the highest risk for dementia. Therefore, the aims of the current systematic review and meta-analysis are (1) to perform a synthesis of existing research of multimodal interventions on cognition and mood for individuals who meet the criteria of MCI and (2) to investigate the clinical implications and limitations of these results for future treatment planning.

## 2 Methods

### 2.1 Eligibility criteria

The eligibility criteria are consistent with the PICO criteria and the PRISMA 2020 reporting guidelines (Page et al., 2021a), and incorporate participants, interventions, comparators, and outcomes. Only RCTs were included in the review with no restrictions on cohort studies, longitudinal studies, and crossover designs.

#### 2.1.1 Participants

Participants included patients with a clinical diagnosis of MCI due to any underlying etiology (e.g., MCI due to AD or Parkinson’s disease), regardless of age, gender, or cultural background. Samples of mixed MCI and healthy or demented older adults were excluded unless an independent analysis was undertaken to evaluate the effect on pwMCI. Because cognitive impairment with no dementia (CIND) was commonly used interchangeably with MCI, participants with CIND were also included. In addition, the Diagnostic and Statistical Manual of Mental Disorders (DSM-5) (American Psychiatric Association, 2013) introduced the term mild neurocognitive disorder (mNCD) to describe acquired cognitive impairments of all causes at all ages before proceeding to identify the etiology. In mNCD, individuals can report slight difficulty performing everyday activities while remaining functionally independent and demonstrate deficits in one or more cognitive domains, which corresponds to MCI symptoms. Therefore, patients with mNCD were also included in the review. However, prodromal AD or other cognitive states (e.g., a score below certain AD risk scales) were excluded due to the potential inconsistency when compared to pwMCI.

#### 2.1.2 Intervention

Intervention eligibility criteria included multimodal behavioral or cognitive interventions to delay or prevent dementia in pwMCI. Any combination of behavioral or cognitive intervention with a pharmacological treatment was excluded unless it was used to compare with a nonpharmacological intervention program. Elective surgical procedures, such as deep brain stimulation, were also excluded. In addition, interventions with variations of the same treatment type (e.g., different physical exercises) were not considered multimodal and excluded. While studies with no cognitive or behavioral interventions or treatment were excluded, a combination of both cognitive and behavioral interventions was not required for inclusion. For example, cognitive training and cognitive rehabilitation were defined as two independent training methods that serve distinctive purposes in patients with dementia (Clare et al., 2003). Specifically, cognitive training consists of guided practice on tasks targeting particular cognitive functions while cognitive rehabilitation focuses on strategies compensating for functional difficulties in daily life. Therefore, interventions with cognitive training and compensatory rehabilitation were included. In a previous systematic review, Gavelin et al. (2021) introduced the concept of exergaming, which referred to video games that provided simultaneous training of different modalities (e.g., cybercycling, a videogame that requires both cycling and navigation strategies). Studies with exergaming were included if multiple modalities were identified.

#### 2.1.3 Comparator

Eligible comparators included nontreatment control groups and alternative multimodal or single modality treatment. However, a comparative effective analysis that aims to investigate the effect of one single intervention arm by adding or withdrawing one of the arms from a multimodal program was excluded due to the lack of appropriate comparison to demonstrate the effect of the overall intervention program. In addition, a direct comparison between targeted multimodal intervention programs and a control group or a group with completely different treatments was required for data extraction.

#### 2.1.4 Outcome measures

To synthesize outcome domains, we referenced two patient-related latent factors derived from our multimodal intervention trial (Smith et al., 2017). Using exploratory factor analysis, Defeis et al. (2021) suggested that common outcome measures in behavioral and cognitive intervention programs for pwMCI could be synthesized into a three-factor model that consisted of patient impairment, patient adjustment, and partner adjustment. This model has been examined and confirmed in a separate MCI intervention sample with high factor loadings and an almost identical structure (Defeis et al., 2021). Therefore, to evaluate the effects of multimodal interventions on patients, the primary outcomes of the current study were organized into patient impairment and patient adjustment categories with their highest loading and most assessed items—cognition and mood. While the quality of life and independent daily functioning outcomes were initially assessed, these outcomes were dropped due to the insufficient number of reports (*k* < 6) and low statistical power.

### 2.2 Information sources

This review only included published studies and abstracts written in or translated into English. PubMed, Embase and Cochrane Library were searched for articles published before January 1st, 2024. In addition, references from relevant publications and symposiums were examined and manually searched as an additional source of literature. Please see Supplementary material B for searching items.

#### 2.2.1 Data management

Search results were imported into Mendeley Reference Manager (Mendeley Support Team, 2011), a software that allows the references to be saved in separate collections and compared for duplicates. The results were then imported to Covidence (Veritas Health Innovation, 2017), an online software with live updates of the collaborative progress and discrepancy for screening and data extraction. Two authors (GY and APL) independently reviewed and evaluated all the records and data in the software.

### 2.3 Data collection process

Targeted variables and measures were identified and extracted by GY and APL independently to an Excel spreadsheet and compared to ensure no errors. Outcomes included changes in cognition and mood. Outcomes were identified by searching the specific terms in the report regardless of measuring tools. Authors were not contacted when information regarding the primary outcome was not available in the text.

### 2.4 Data items

Participant age, study attrition rate, diagnostic criteria, specific multimodal intervention strategies and characteristics (duration, frequency, and follow-up duration), comparator characteristics (no treatment vs. alternative treatment), outcome measures, effect sizes for each outcome, and results reported by the authors were extracted and documented for all eligible publications.

### 2.5 Risk of bias in individual studies

**Figure 1:**
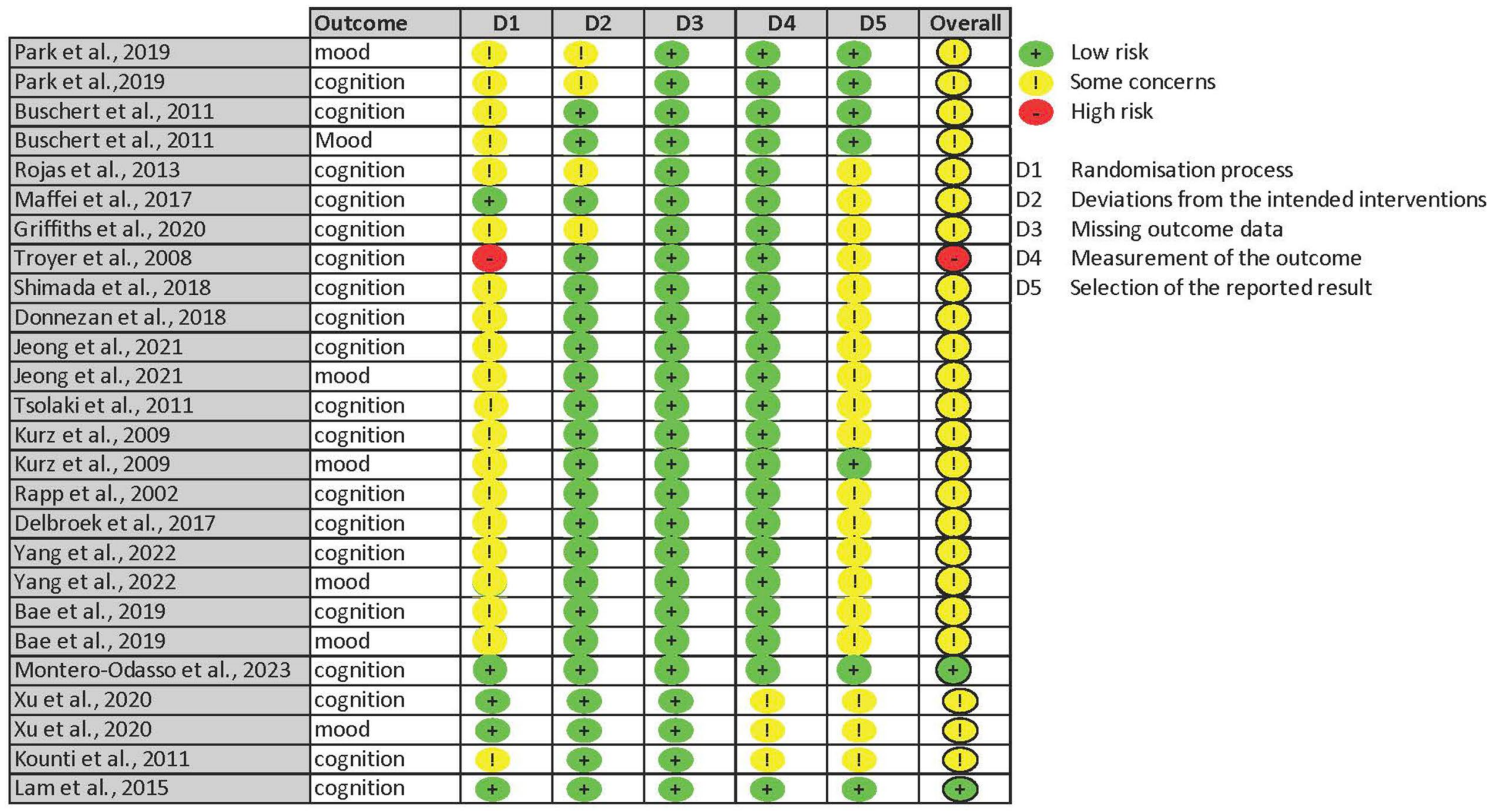
Risk of bias traffic plot.

The revised Cochrane Collaboration software (RoB 2) (Sterne et al., 2019) assessing the risk of bias in RCTs was employed in the current review. Detailed criteria of focus in each domain can be found in the Cochrane Handbook Chapter 8.2 (Higgins et al., 2019). An overall risk-of-bias judgment was obtained for individual domains by both GY and APL. Similarly, a consensus meeting was arranged to resolve any discrepancies during the process. Results of the risk-of-bias assessment were then visualized through another web-based R package, *robis* (McGuinness and Higgins, 2021). Because several studies included both targeted outcomes, each outcome was assessed separately and weighted equally in the evaluation. Figure 1 depicts the results of 26 parallel design evaluations conducted for 15 clinical trials.

Comparisons of the baseline characteristics were employed to evaluate any effects raised by the randomization process. Studies that failed to report any differences between the intervention and control groups regarding demographic variables (e.g., age, gender, etc.) or targeted outcomes (e.g., cognition) raised concern about whether an appropriate analysis was used to estimate the effect of assignment (Domain 2) and whether baseline differences suggested a problem with randomization (Domain 1). “No information” on the randomization process (Domain 1) was given to a few studies, which led to a rating of “some concerns,” due to a failure to clarify the sequence allocation method. In addition, “probably no” was given to one study using consecutive recruitment with no information on the randomization strategy (Kurz et al., 2009). Studies with a larger than 5% dropout rate, according to the guidelines, were rated as “probably not” for whether the outcomes were provided for almost all the participants (Domain 3). If the reasons for attrition were provided and were irrelevant to participants’ cognitive functioning, the overall rating for the domain remained “low risk.”

### 2.6 Meta-analysis

The goal of a meta-analysis is to estimate the overall effect of treatments across studies. However, because studies vary in the quantity and quality of information, different weight is assigned to each study (e.g., higher weight assigned to larger studies) to calculate a combined effect. Due to the variability of sample sizes and characteristics among the reports included in the current study, we used the random effect model of meta-analysis, which assumes that each study is estimating a different effect size, to estimate the mean of a distribution of true effects for each outcome.

#### 2.6.1 Effect measures

Effects sizes were assessed through standardized mean differences (SMDs) estimated by Hedge’s *g*, which is less biased by small sample sizes compared to Cohen’s *d* (Hedges, 1981; Lin and Aloe, 2021). Similar to Cohen’s *d,* Hedge’s *g* visualizes effects by separating them into multiple levels: small (0–0.2), small-to-medium (0.2–0.5), medium-to-large (0.5–0.8), and large effects (>0.8). Hedge’s *g* was collected as the primary effect measure when available or calculated manually when it was not originally reported. The following formula was employed for the calculation: $g=\frac{\left(\mu ,-\mu_{2}\right)}{\sqrt{\left(\left(n_{1}-1\right)s_{1}^{2}+\left(n_{2}-1\right)s_{2}^{2}\right)∕\left(n_{1}+n_{2}-2\right)}}$ , where μ denotes the changes in mean during the time frame, *s* denotes the standard deviation of change for each group, and *n* stands for the sample size of each group. Change from baseline standard deviation was imputed through the following formula extracted from the Cochrane Handbook (Higgins, 2008): $\begin{array}{l} SD_{E,\mathrm{change}}\\ =\sqrt{SD_{E,\mathrm{baseline}}^{2}+SD_{E,\mathrm{final}}^{2}-\left(2\times \mathrm{Corr}\times SD_{E,\mathrm{baseline}}\times SD_{E,\mathrm{final}}\right)} \end{array}$ , where *Corr* was calculated using the following steps from studies with available change-from-baseline standard deviation for the same measure. To calculate the *Corr* for a specific outcome measure, we obtained (1) the correlation for the experimental group $Corr_{E}=\frac{SD_{E,\mathrm{baseline}}^{2}+SD_{E,\mathrm{final}}^{2}-SD_{E,\mathrm{change}}^{2}}{2\times SD_{E,\mathrm{baseline}}\times SD_{E,\mathrm{final}}}$ , (2) the correlation for the control group, and finally (3) using each correlation to obtain the standard deviation of change for each group. In studies with only Cohen’s *d*, bias-correction was applied: *g*= $\frac{d}{\sqrt{N∕df}}$ =(1–3/(4*(*n*_1_ + *n*_2_–2) − 1)) × *d* (originally from Hedges, 1981 but later adjusted by Borenstein et al., 2009). For studies with solely F-statistics, *g* was calculated using the R package *ESC* (Lüdecke et al., 2019). Due to the heterogeneity and dependency of effects among measurements in the cognitive domain, a multilevel meta-analysis was performed. Specifically, results for each outcome measure (level 1) were clustered by study (level 2) to create a pooled effect size for each study (level 3). Aggregated effect sizes and confidence intervals were then calculated through the between and within cluster variances via the R package *Metafor* (Harrer et al., 2021).

**Figure 2:**
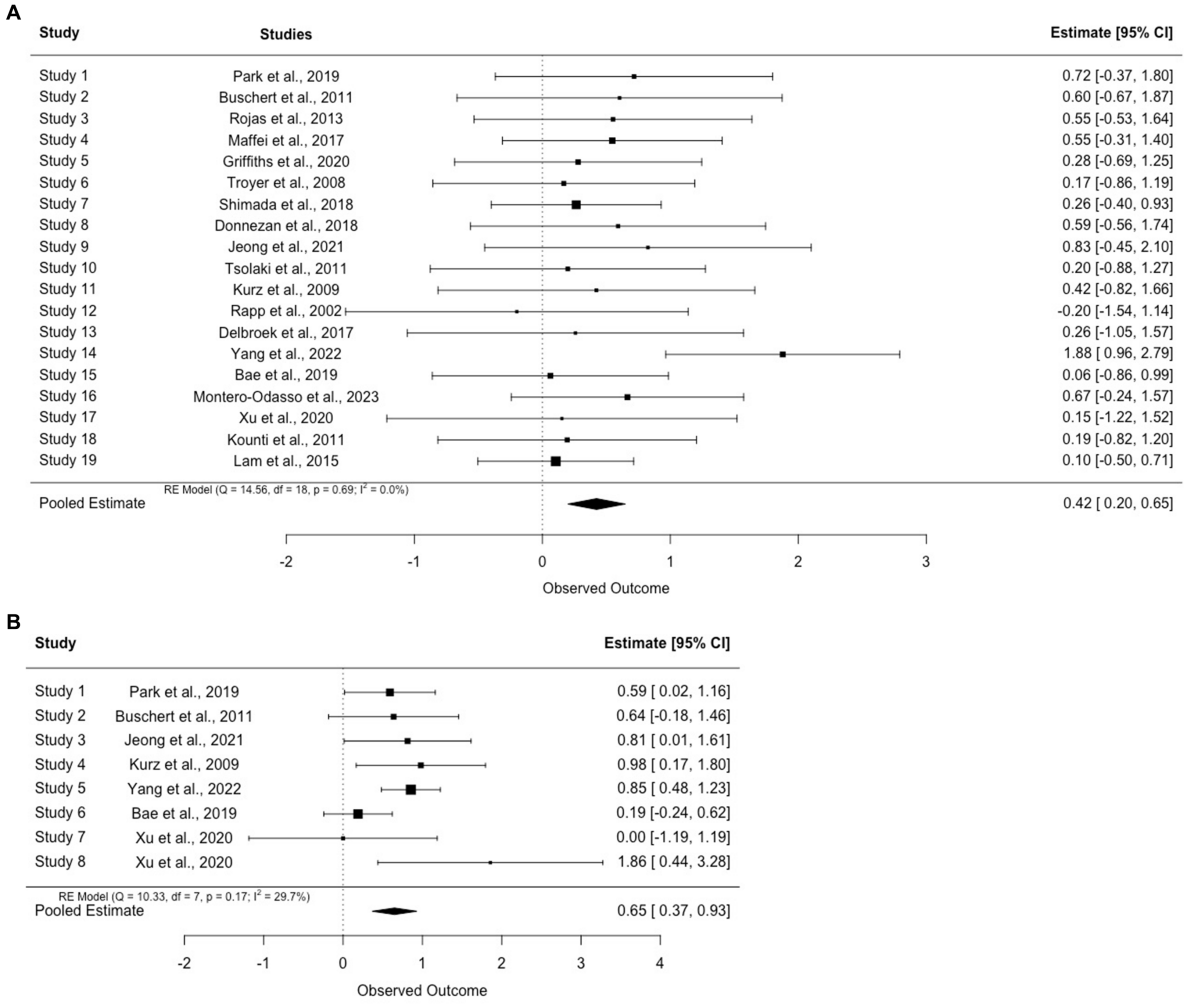
**(A)** Forest plot for cognition outcomes. **(B)** Forest plot for mood outcomes.

Results were reported primarily via changes from baseline or group-by-time interactions to indicate different trajectories between groups. Effect sizes were calculated manually for most outcomes by the primary reviewer (GY) to reflect between group differences in changes and to perform standardized comparisons among studies. An average effect size was employed for cognition in each report due to the heterogeneity of assessments. Because higher scores on the Alzheimer’s Disease Assessment Scale-Cognitive Subscale (ADAS-cog) and the Trail Making Test (TMT) reflect greater impairment, changes in these scales were reversed during calculation. For mood outcomes, score changes were reversed for anxiety/depression outcomes. General study and intervention characteristics are summarized in Table 1. A summary of intervention components, which were synthesized into physical exercise, social skills, cognitive training, cognitive stimulation, and others, is presented in Table 2. Results and measures were synthesized into different outcomes and factors and are presented in Table 3 and Figures 2A,B.

**Table 1:** Characteristics of multimodal intervention studies for patients with mild cognitive impairment.

	Country	Dx	Diagnostic criteria	Age	Attrition (%)	I (N)	C(N)	Intervention	Comparator	Length	Frequency	Follow-up duration
Park et al. (2019)	Korea	aMCI	Clinical interview by a dementia specialist, neurological examinations, blood test, brain computed tomography/MRI, and detailed neuropsychological assessments.	71.63	8.16	25	24	Dual-task trainings that consisted of cognitive and exercise tasks	Untreated control	24 weeks	Weekly sessions	Week 12 and 24.
Buschert et al. (2011)	Germany	aMCI	Petersen et al. (2001)	71.20	5.13	12	12	Cognitive training of memory function, cognitive stimulation, reminiscence discussions, and group psychomotor and recreational tasks.	Paper-pencil exercises and monthly meetings.	6 months	20 weekly 120-min sessions	End of the intervention
Rojas et al. (2013)	Argentina	MCI	Petersen et al. (1999); Petersen et al. (2001); neurological examinations, routine laboratory analyses, and brain CT/MRI	74.46	34.78	24	22	Cognitive stimulation with episodic memory encoding and executive control training, cognitive training with theoretical strategies and external aids (e.g., calendar)	Routine treatment with monthly consultations with their doctor.	6 months	120-min 2x/week	6-months after the intervention
Maffei et al. (2017)	Italy	MCI	The European Consortium on Alzheimer’s Disease Working Group on MCI.	74.50	8.85	55	58	Cognitive stimulation, social games, multimedia computer exercises, music therapy, movie watching and discussion, paper and pen tests, and aerobic exercise training.	Untreated control	7-month	8 cycles of 18 60-min sessions, 3x/day for 3x/week, every other day from Monday to Friday. Each cycle was completed within 3 weeks.	End of the intervention & 12 months after the intervention
Griffiths et al. (2020)	Thailand	mNCD/MCI	MoCA and DSM-5 by a specialist geriatrist or a neurologist	NA	0	35	35	Physical movement using bamboo with music, operational therapy, and multifaceted cognitive training.	Untreated control	12 weeks	2x/week	End of the intervention
Shimada et al. (2018)	Japan	MCI	Subjective memory complaints on questionnaires and age-adjusted scores >1.5 SD below the mean on any cognitive test but were functionally independent in basic ADL.	71.60	13.6	154	154	Dual-task training that combined physical and cognitive tasks	Health promotion classes in health education	40 weeks	90-min weekly sessions	End of the intervention
Donnezan et al. (2018)	France	MCI	Determined by a neuropsychologist with evidence of executive deficits	76.80	2.86	21	15	Simultaneous physical and cognitive training: aerobic training on bikes and cognitive training using 33 preselected games to stimulate attention, working memory, mental flexibility, inhibition, reasoning and updating.	Usual lifestyle with no novel physical activity or cognitive stimulation.	12 weeks	1-h sessions 2x/week	End of the intervention & at 6-months
Jeong et al. (2021)	Korea	aMCI	Park et al. (2017), and clinical interview by a dementia specialist.	71.00	13.33	13	13	Physical activity promotion, behavior modification, and multi-task programs involving cognitive and exercise tasks.	Monthly educational classes	12 weeks	90-min session 2x/week.	End of the intervention
Tsolaki et al. (2011)	Grace	MCI	Petersen et al. (2001), Artero et al. (2006)	67.82	12.43	104	72	Cognitive training, cognitive stimulation, and cognitive-behavioral psychotherapeutic techniques.	Waitlist	6 months	90-min sessions 3x/week	End of the intervention
Kurz et al. (2009)	Germany	MCI	> 1.5 SD below the age and education norm on >1 domain of the CERAD neuropsychological battery, had declined from a previously higher cognitive level according to an informant, showed little or no limitations on complex ADL, and CDR = 0.5.	70.56	0	18	10	Practical problem-solving and self-assertiveness training, relaxation techniques, stress management, cognitive training, and motor exercises. Weekly information and support group for caregivers.	Waitlist	4 weeks	Weekdays from 9:00–15:00	End of the intervention
Rapp et al. (2002)	United States	MCI	Petersen et al. (1999)	75.21	15.79	9	10	Dementia information, relaxation, and memory skills (cueing, categorization, chunking, method of loci) training	Untreated control	6 weeks	2-h weekly sessions	End of the intervention
Delbroek et al. (2017)	Belgium	MCI	MOCA<26	87.2	15	10	10	Dual tasks that involve memory exercise and avoidance whilst walking.	Usual care in the nursing home	6 weeks	18–30 min 2x/week.	End of the intervention
Yang et al. (2022)	China	MCI	Petersen et al. (1997)	70.19	8.2	61	61	Dietary intervention, physical training, and computerized cognitive training	Usual care	6 months	Dietary intervention: six 10–30 min meetings 1x/3–4 weeks; physical exercise: 1x/week for the first month and 2x/week for the remaining months; cognitive training: 1x/week for 60–90 min	1-, 3-, and 6-months after the intervention
Bae et al. (2019)	Japan	MCI	> 1.5 SD below the age-and education-adjusted score for >1 cognitive domains, MMSE ≥24, no need for supervision or external assistance in performing basic ADL, no dementia.	75.96	32.53	41	42	Physical, cognitive, and social activities.	Two 90-min health education classes on oral care and nutrition.	6 months	Sixteen 90-min sessions for each activity (48 in total), 2x/week	End of the intervention
Montero-Odasso et al. (2023)	Canada	MCI	Subjective cognitive concerns, objective impairment in memory, executive function, attention, and/or language, preserved activity of daily living, no dementia.	73.09	22.74	69	34	Exercise and cognitive intervention	Balance-toning exercise, sham cognitive training, and placebo vitamin D	20 weeks	90-min sessions 3x/week	End of the intervention
Xu et al. (2020)	China	MCI	Scored 19–21 after adjusting for years of educational (+1 point if <6 years) on the Montreal Cognitive Assessment Hong Kong version (HK-MoCA)	74.00	8.33	6	5	Cognitive training (Rummikub) and Taichi	Health advice	12 weeks	60-min of cognitive training and 30 min of Taichi 3x/week	3-month and 6-month
Kounti et al. (2011)	Greece	MCI	Petersen et al. (2001); Artero et al. (2006)	69.16	34.09	29	29	RHEA: visuomotor, and verbal-kinetic dual tasks	Waitlist	20 weeks	90-min 1x/week	6-month
Lam et al. (2015)	China	MCI	International Working Group on Mild Cognitive Impairment	75.85	26.24	132	131	One cognitive and two types of mind–body exercise	Social activities	12-month	1-h of each training	4-, 8-, 12-month

Dx, diagnosis; I, Intervention; C, Comparator; MCI, mild cognitive impairment; aMCI, amnestic MCI; mNCD, mild neurocognitive disorder; SD, standard deviation; MRI, magnetic resonance imaging; CT, computed tomography.

**Table 2:** Multimodal intervention components.

	Physical training									Social skills			Cognitive training				Cognitive stimulation			Other			
	Aerobic exercise	Muscle strength training	Postural balance	Physical activity promotion	Behavior modification	Psychomotor exercise	Recreational exercise	Unspecified motor training	Mind–body exercise (Taichi)	Social interaction exercise	Working memory/attention	Visuospatial skills	Memory	Executive function	Semantic abilities	Demanding leisure activities/games	Compensatory techniques	Metacognition/cognitive self-efficacy	Activation of everyday life activities	Music therapy	Operational therapy	Lifestyle/dementia information	Psychotherapy
Park et al. (2019)	✓			✓	✓						✓												
Buschert et al. (2011)						✓	✓			✓	✓	✓	✓				✓		✓				
Rojas et al. (2013)										✓			✓	✓			✓	✓					
Maffei et al. (2017)	✓									✓	✓		✓	✓	✓					✓			
Griffiths et al. (2020)	✓										✓		✓	✓					✓	✓	✓		
Shimada et al. (2018)	✓	✓	✓								✓												
Donnezan et al. (2018)	✓										✓			✓									
Jeong et al. (2021)	✓			✓	✓						✓			✓	✓							✓	
Tsolaki et al. (2011)											✓		✓	✓									✓
Kurz et al. (2009)								✓					✓	✓			✓					✓	✓
Rapp et al. (2002)													✓									✓	✓
Delbroek et al. (2017)											✓		✓									✓	
Yang et al. (2022)	✓	✓	✓								✓	✓	✓	✓	✓								
Bae et al. (2019)	✓	✓								✓	✓												
Montero-Odasso et al. (2023)	✓	✓											✓										
Xu et al. (2020)									✓							✓							
Kounti et al. (2011)		✓	✓					✓			✓	✓	✓	✓	✓								
Lam et al. (2015)	✓	✓							✓							✓							

**Table 3:** Summary of findings of multimodal interventions on primary outcomes.

Studies	Measures	*g*	Results
**Cognition**			
Park et al. (2019)	Modified ADAS-cog, DST, DSST, and K-MMSE	0.72	The intervention group exhibited a significantly improved modified ADAS-cog score (*p* < 0.01), working memory (*p* = 0.02), and executive function scores (*p* < 0.01)
Buschert et al. (2011)	ADAS-cog, MMSE, TMT A & B, and RBANS story memory & story recall	0.60	A significant interaction between treatment and progression was found for ADAS-cog (*F*(1,18) = 6.2, *p* = 0.02, η^2^ = 0.26), MMSE (*F*(1,18) = 3.8, *p* = 0.07, η^2^ = 0.17), RBANS-story memory (*F*(1,18) = 3.4, *p* = 0.08, η^2^ = 0.16), and TMT-B (*F*(1,18) = 3.5, *p* = 0.08, η^2^ = 0.16). Main effects were found for MMSE (*F*(1,18) = 8.5, *p* < 0.01, η^2^ = 0.23) and RBANS story memory (*F*(1,18) = 12.5, *p* < 0.01, η^2^ = 0.41) and recall (*F*(1,18) = 9.9, *p* < 0.01, η^2^ = 0.36).
Rojas et al. (2013)	MMSE, CDR, Signoret’s Memory Battery, BNT, Verbal Fluency, WAIS vocabulary, similarities, matrix reasoning, & block design, TMT A & B, and WAIS-III DSF and DSB	0.55	In the control group, significant differences were found in MMSE (*p* < 0.002, mean change = 1.77), CDR (*p* < 0.02, mean change = −0.1), recognition (*p* < 0.05, mean change = 1.29), and semantic fluency (*p* < 0.01, mean change = 2.40). Conversion to dementia was seen in 1 trained and 3 non-trained patients at the 12-month follow-up. The trained group improved on the BNT (mean change = −2.87, *p* = 0.04) and semantic fluency (mean change = −3.03, *p* < 0.01).
Maffei et al. (2017)	ADAS-cog	0.55	A significant beneficial effect of the intervention on ADAS-cog was detected over time (difference = −2.17, *p* < 0.001, 95% CI (−0.60, 0.50))
Griffiths et al. (2020)	TMT-A & B, DST, the Verbal Fluency Test, the wordlist learning test, and Block Design	0.17	Significant improvement was seen in DST (*p* = 0.024), letter and category fluency (*p* = 0.001 and *p* = 0.004, respectively), and immediate and delayed recall (*p* = 0.001 and *p* = 0.001, respectively). Significant differences were found in immediate and delayed recall (*p* = 0.023 and *p* = 0.036, respectively). Only the intervention group improved in executive function (*p* = 0.029).
Shimada et al. (2018)	MMSE, WMS-R LM II, RAVT, verbal fluency letter and categorical test, and TMT	0.26	Compared with the controls, the intervention group exhibited significantly greater score changes on the MMSE (difference = 0.8, *p* = 0.012), WMS-LM II (difference = 1.0, *p* = 0.004), letter fluency (difference = 3.6, *p* < 0.001), and category fluency (difference = 2.2, *p* = 0.002) tests, but not the RAVLT (difference = 0.2, *p* = 0.352) or the TMT (difference = 0.4, *p* = 0.350).
Donnezan et al. (2018)	Matrix Reasoning, the flexibility part of the Stroop Color Word test, DSF, and DSB	0.59	Performance was improved on the Matrix Reasoning (*p* < 0.001) post-intervention. Improvement was observed in DSF (*p* < 0.01) and DSB (*p* < 0.001) immediately and at 6-month (DSF: *p* < 0.001; DSB: *p* = 0.0) post-intervention.
Jeong et al. (2021)	K-MMSE, Modified ADAS-cog, TMT A & B, and DSST	0.83	In the intervention group, modified ADAS-cog (*p* < 0.05), mean TMT-A (*p* < 0.01), and mean TMT-B (*p* < 0.01) significantly decreased, while mean DSST (*p* < 0.01) significantly increased. A significant group by time interaction was shown in mean TMT-A (*p* < 0.05), mean TMT-B (*p* = 0.01), and mean DSST (*p* = 0.02).
Tsolaki et al. (2011)	MMSE, MoCA, RBM, RAVLT, RCFT, TEA, DSST from WAIS-R, FUCAS, TMT-B, Verbal Fluency Test-FAS, BNT, and BDAE	0.20	Executive function (*p* = 0.004), verbal memory (*p* = 0.003), visual-constructive abilities (*p* < 0.012), and general cognitive performance (*p* < 0.005) improved post-intervention.
Kurz et al. (2009)	MMSE, CVLT, and RCFT	0.42	Both verbal and non-verbal memory scores improved (*p* < 0.001) in the intervention group but not in the waitlist control.
Rapp et al. (2002)	CERAD and four memory tasks (a word list, a grocery list, names and faces, and paragraphs).	−0.20	No significant differences were found between groups in memory performance. The trained group had higher scores on the wordlist task delayed recall than the control group (*p* = 0.08, *R*^2^ = 0.11).
Delbroek et al. (2017)	MoCA	0.26	No changes were detected over time for either group.
Yang et al. (2022)	MoCA	1.88	Significant improvement was detected in the intervention group over three time points (Wald ꭕ^2^ (3) =303.928, *p* < 0.01).
Bae et al. (2019)	NCGG-FAT delayed word list recall and immediate recognition, Corsi block-tapping task, TMT A & B, and MMSE	0.06	The intervention group had significantly greater improvements in spatial working memory (*p* = 0.024) compared with the control group. However, MMSE, composite word memory, TMT-A, TMT-B, and SDST scores showed no significant between-group differences following the intervention.
Montero-Odasso et al. (2023)	ADAS-cog 13	0.67	A combination of exercise regime and cognitive training significantly improved the ADAS-cog-13 compared with the active control (mean difference = −2.52, 95%CI = [−4.09, −0.94], *p* = 0.002).
Xu et al. (2020)	ADAS-cog and MOCA	0.15	No group x time interaction was noted favoring the cognitive-physical intervention group.
Kounti et al. (2011)	MMSE, FUCAS, WSCT, TEA, WAIS-R, RAVLT, RCFT, BNT, and verbal fluency	0.19	The intervention group differed from the control in changes in general cognitive performance (MMSE) (*p* = 0.047), speed of selective visual attention (TEA) (*p =* 0.002), visuospatial constructional (copying) abilities (ROCFT-C) (*p =* 0.013) and verbal fluency (FAS) (*p =* 0.015).
Lam et al. (2015)	ADAS-cog, CMMSE, delayed recall, CVFT	0.10	The integrated cognitive and physical exercise group showed greater improvements in CVFT (time × intervention effects, χ^2^ = 23.38, *p* < 0.001).
**Mood**			
Park et al. (2019)	SGDS-K	0.59	The intervention group exhibited significantly improved depressive symptoms (*p* = 0.02).
Buschert et al. (2011)	MADRS	0.64	A significant group by time interaction was found (*F*(1,18) = 8.8, *p* < 0.01, η^2^ = 0.33).
Jeong et al. (2021)	SGDS-K	0.81	A significant group-by-time interaction was shown (*p* = 0.01)
Kurz et al. (2009)	BDI	0.98	The depression score lowered by 50% (*p* < 0.01) in the intervention group but not in the waitlist control
Yang et al. (2022)	GDS	0.85	Significant improvement was detected in the intervention group over three time points on depressive symptoms (Wald ꭕ^2^ (3) = 126.102, *p* < 0.01)
Bae et al. (2019)	GDS	0.19	No observed differences in GDS score.
Xu et al. (2020)	GDS-15 and GAS	1.86	Group × time interaction was found to favor the cognitive-physical intervention group (*p* = 0.026) over nurse-led risk factor modification and health advice in reducing anxiety but not depressive symptoms.

ADAS-cog, Alzheimer’s Disease Assessment Scale-Cognitive Subscale; DST, Digit Span Test; DSST, Digit Symbol Substitution Test; RBANS, Repeatable Battery for the Assessment of Neuropsychological Status; MMSE, Mini-Mental Status Exam; K-MMSE, Korean version of MMSE; CMMSE, Chinese version of MMSE; TMT, Trail Making Test; RBM, Rivermead Behavioral Memory Test; CDR, Clinical Dementia Rating; BNT, Boston Naming Test; WAIS, Wechsler Adult Intelligence Scale; DSF, Digit Span Forward; DSB, Digit Span Backward; MoCA, Montreal Cognitive Assessment; WMS-R, Weschler Memory Scale-Revised; LM, Logical Memory; RAVLT, Rey Auditory Verbal Learning Test; RCFT, Rey-Osterrieth Complex Figure Test-Delayed Recall; TEA, Test of Everyday Attention; WAIS-R, WAIS Revised; FUCAS, Functional Cognitive Assessment Scale; BDAE, Boston Diagnostic Aphasia Examination; CVLT, California Verbal Learning Test; CERAD, Consortium for the Registry of Alzheimer’s Disease; RAVLT, Rey Auditory Verbal Learning Test; CVFT, category verbal fluency test; NCGG-FAT, National Center for Geriatrics and Gerontology-Functional Assessment Tool; GDS, Geriatric Depression Scale; SGDS-K, Korean version of the short GDS; MADRS, Montgomery-Asberg Depression Rating Scale; BDI, Beck Depression Inventory; GAS, Geriatric Anxiety Scale.

According to the AMSTAR 2 guidelines, studies with a high risk of bias were excluded from the meta-analysis. The overall systematic review and meta-analysis were rated as “high quality” in AMSTAR 2 (Shea et al., 2017).

### 2.7 Heterogeneity

Between-study variance, Ƭau^2^, was calculated through total variance (Cochrane’s Q), which denotes the squared deviations of each study from the combined mean, and the degrees of freedom (df). Due to the small sample size and heterogeneity across study populations, the random effects model with maximum likelihood (Borenstein et al., 2007) was employed to compute the heterogeneity and combined effect of the studies. For cognition, the aggregated model was used to indicate heterogeneity attributed to the variance across studies. In addition, to account for the impact of sample size on Q, we calculated the total proportion of variance owing to heterogeneity (I^2^) for each outcome (Higgins et al., 2013). In general, I^2^ categorizes results into low (25%), moderate (50%), or substantial (75%) heterogeneity. The analyses were performed on *Metafor* (Viechtbauer, 2010).

### 2.8 Publication bias

Publication bias generally refers to the probability of bias stemming from unpublished results of studies with non-significant data (Borenstein et al., 2009). A common way of assessing publication bias is through the level of symmetry of a funnel plot, which depicts the relationship between effect sizes and standard error in each study. Because small studies are more likely to generate non-significant results and have a larger standard error, they are less likely to be published. The funnel plot inverted the y-axis (standard error) to position these smaller studies at the bottom while placing the larger ones on the top. Thus, the top of the funnel should distribute closely to the mean effect size whereas the bottom should scatter heavily on both the left and right sides (the shape of a funnel) when there is no publication bias. Aside from the graph, we also used the modified Egger’s regression test by Pustejovsky (Egger et al., 1997; Pustejovsky and Rodgers, 2019) to assess asymmetry of the funnel plots incorporating the standard error of between group SMD using the following formula: $SE_{*SMD_{\mathrm{between}}}=\sqrt{\frac{n_{1}+n_{2}}{n_{1}n_{2}}}$ . The resulting value is equivalent to a z-score with a similar rejection range above 1.96 or below −1.96 for a significance level below 0.05. These tests were all performed through *Metafor* and *Dmetar* (Viechtbauer, 2010; Harrer et al., 2021) in R (R Core Team, 2014).

## 3 Results

### 3.1 Study selection

A total of 482 results were identified after a systematic search of PubMed (*k* = 126), Embase (*k* = 106), and Cochrane Library database (*k* = 250). Among them, 105 duplicates were removed prior to screening, which yielded 377 results for review. A preliminary abstract/title review excluded 356 articles, of which the majority were study protocols or interventions targeting combined MCI and dementia populations. In the remaining 21 reports, 10 were excluded after a full-text review. A list of excluded reports was provided in Supplementary material C. Specifically, three studies were excluded due to a lack of multimodal intervention. Two studies used comparative effectiveness analysis. In addition, four studies were excluded because the group receiving multimodal interventions was not directly compared to the double-sham control group but to other single-modal interventions, and one study lacked randomized groups. In the end, 11 clinical trials were included from the databases for review.

**Figure 3:**
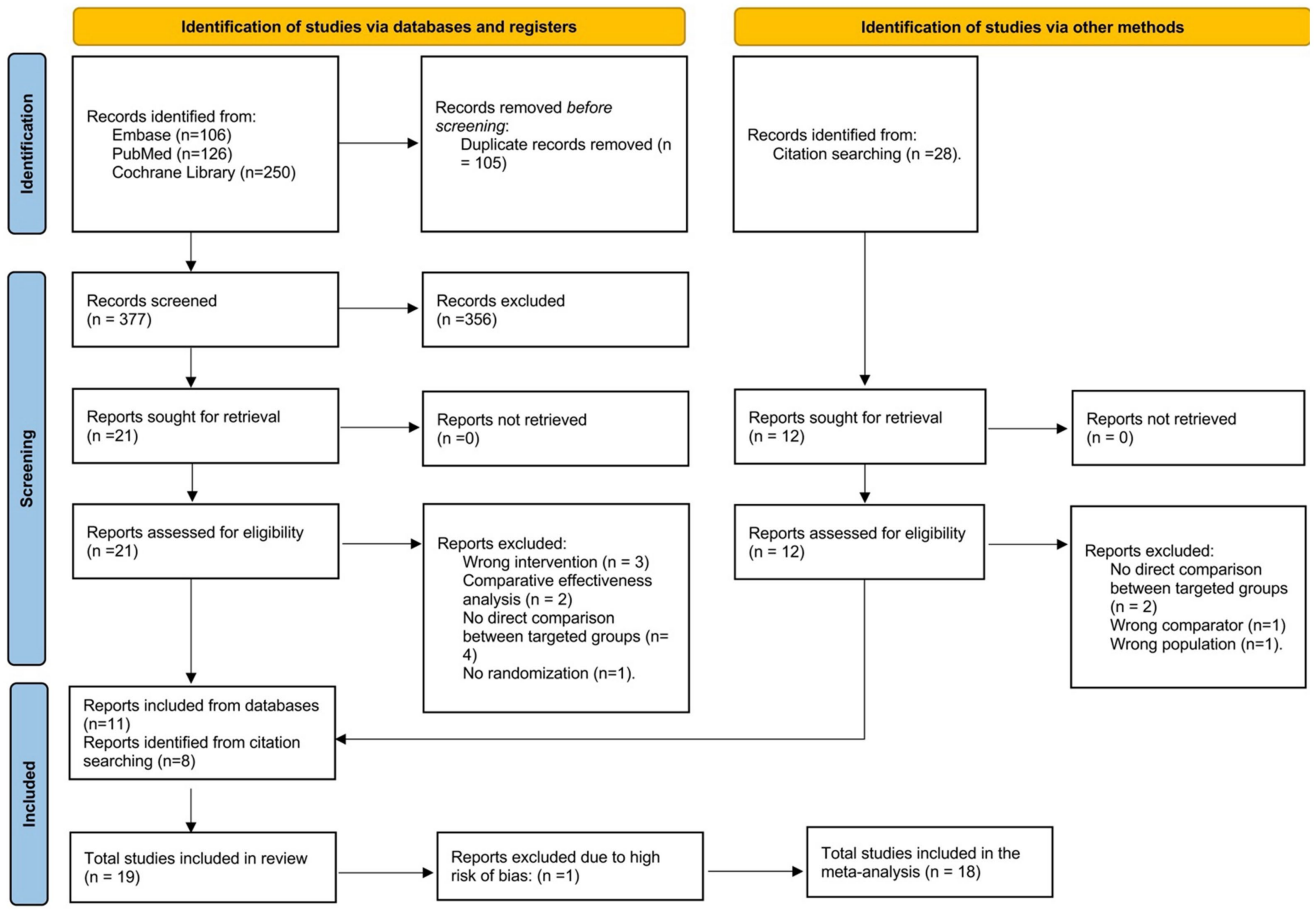
PRISMA flow diagram.

Manual citation searching from previous literature reviews (Chandler et al., 2016; Karssemeijer et al., 2017; Gheysen et al., 2018; Gavelin et al., 2021; Han et al., 2022; Meng et al., 2022) found 28 results that did not overlap with the primary database search. After abstract/title screening, 12 remained for full-text screening. Of those clinical trials, two multi-group studies with no direct comparison between the multimodal and control groups, one study with a wrong comparator (i.e., the control group received mixed interventions), and one report with a mixed sample of MCI and dementia patients were removed. As a result, eight studies were included in the final review. A detailed PRISMA 2020 flowchart is demonstrated in Figure 3 (Page et al., 2021b).

### 3.2 Overview

Overall, 19 journal articles were eligible for the final review, and 18 were included in the meta-analysis. One report (Troyer et al., 2008) was excluded due to the high risk of bias (Figure 1). Of these, 18 reports of cognition (*n* = 1,555, mean age = 73.54 years old) and seven reports of mood (*n* = 343, mean age = 72.08 years old) were identified. A few reports failed to include an effect size or a *p* value for nonsignificant results, for which certain outcomes were not included in data extraction.

Participants’ mean ages were obtained from baseline characteristics for most of the studies except for Griffiths et al. (2020), which only included the number of participants in two age groups (60–69 years) and (70–79 years). Mean ages ranged from 67.82 to 87.20 with a standard deviation of 4.26. The attrition rate ranged from 0 to 34.78% with three studies (Kounti et al., 2011; Rojas et al., 2013; Bae et al., 2019) reporting above 30% dropout rates at the end of the intervention. Details regarding age, attrition rate, intervention methods, sample size, country, and follow-up durations are presented in Table 1.

### 3.3 Risk of bias

Some concerns were reported for most of the studies due to the lack of published protocols for a proper comparison between the actual analysis and an analysis plan before unblinded outcome data were available (Domain 5). Other common concerning criteria included whether participants were aware of their assigned intervention during the trial (Domain 2) and whether the allocation sequence was concealed from participants until enrollment (Domain 1). Maffei et al. (2017), Lam et al. (2015), Xu et al. (2020), and Montero-Odasso et al. (2023) were the only studies that explicitly stated that participants were not informed of their group assignment until the beginning of the intervention. Troyer et al. (2008)’s randomization process (Domain 1) was rated “high risk” due to missing information regarding allocation concealment and significant group differences favoring the control group on cognitive functioning at baseline. Therefore, the study was not included in the final meta-analysis. In the end, only two studies (Lam et al., 2015; Montero-Odasso et al., 2023) received an overall rating of “low risk.”

### 3.4 Heterogeneity

In general, heterogeneity was low for mood (Ƭau^2^ = 0.046, Q(7) = 10.33, *p* = 0.17, I^2^ = 29.7%) and minimum-low for the aggregated cognition outcomes (Ƭau^2^ = 0.040, Q(17) = 21.71, *p* = 0.20, I^2^ = 21.7%). Study characteristics such as sample size, education, frequency of intervention, and intervention modalities might serve as potential sources of heterogeneity. Heterogeneity as indicated by I^2^ represented between-study variability regardless of the number of studies. In this case, studies involving either mood or cognition outcomes only differed by sampling error, which did not appear to impact the overall aggregated meta-analysis model.

### 3.5 Publication bias

**Figure 4:**
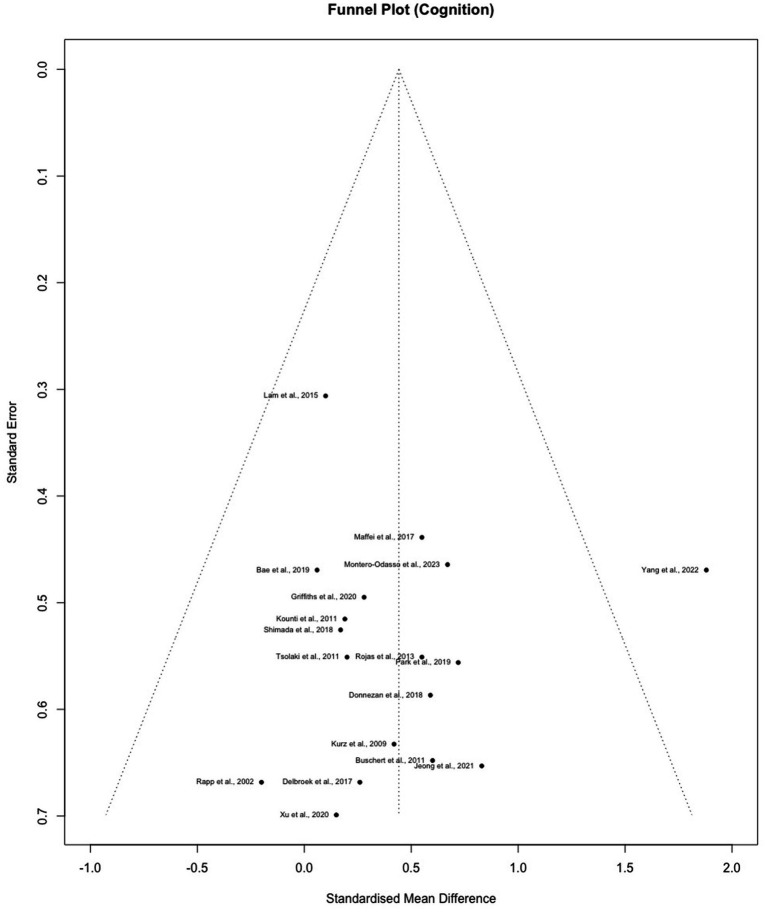
Funnel plot for cognition outcomes.

**Figure 5:**
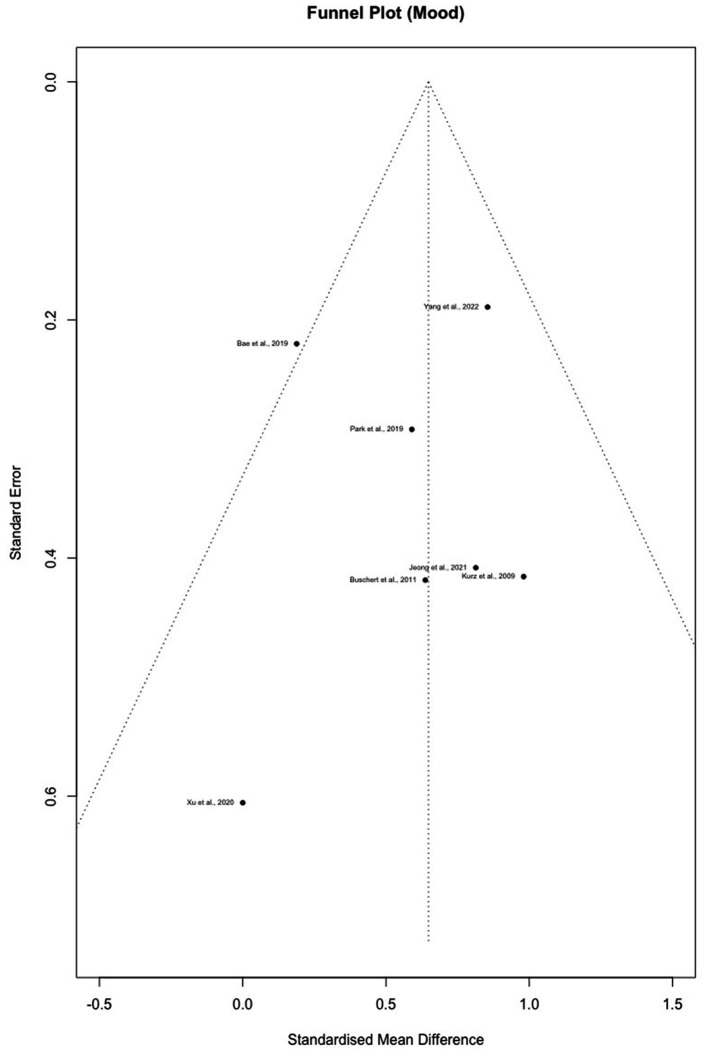
Funnel plot for mood outcomes.

Egger’s test with adjustment did not indicate asymmetry in the funnel plot for cognition (bias = −1.59, intercept =0.62, *t*(16) = −1.29, *p* = 0.216) or mood (bias = 0.46, intercept = 0.51, *t*(6) = 0.37, *p* = 0.727), which reflects the absence of publication bias in both outcomes (Figures 4, 5).

### 3.6 Primary outcomes

#### 3.6.1 Cognition

Overall, the average effect sizes for cognition ranged from −0.20 (Rapp et al., 2002) to 1.88 (Yang et al., 2022). The pooled effect size was small to medium (*g* = 0.44, 95% CI = [0.21–0.67]). Notably, Rapp et al. (2002) and Xu et al. (2020) were the only two studies that reported no differential cognitive improvement between groups. In addition, minimal to small improvement was found in four reports (Kounti et al., 2011; Lam et al., 2015; Bae et al., 2019; Xu et al., 2020), small to medium effect (0.20 < *d* < 0.50) was found in five reports (Kurz et al., 2009; Tsolaki et al., 2011; Delbroek et al., 2017; Shimada et al., 2018; Griffiths et al., 2020), and medium to large effect (0.50 < *d* < 0.80) was reported in six studies (Buschert et al., 2011; Rojas et al., 2013; Maffei et al., 2017; Donnezan et al., 2018; Park et al., 2019; Montero-Odasso et al., 2023). Large effects (*d* > 0.80) were demonstrated in the two latest studies that were both conducted in Asia (Jeong et al., 2021; Yang et al., 2022).

#### 3.6.2 Mood

The pooled effect size for mood was medium to large (*g* = 0.65, 95% CI = [0.37–0.93]). While mood was commonly measured at baseline to examine group balance post randomization, it was not used as an outcome throughout follow-ups. Among all the included studies, depression was the only outcome evaluated post-intervention except for Xu et al. (2020), which demonstrated a higher reduction (*p* = 0.026) in anxiety with multimodal interventions. Notably, the study did not find any benefits of multimodal intervention in reducing depression. Effects sizes ranged from 0 (Xu et al., 2020) to 0.98 (Kurz et al., 2009) for depressive symptoms and large (*g* = 1.86) for anxiety.

## 4 Discussion

The purpose of this systematic review and meta-analysis was to summarize and synthesize results from current literature on the effects of multimodal cognitive and behavioral interventions on cognition and mood for pwMCI. A systematic search of three databases (PubMed, Embase, and Cochrane Library) and reference lists revealed 18 journal articles for the review (Figure 3). Unfortunately, most studies involved some risk of bias according to the RoB2 Cochrane analysis tool for parallel (Figure 1) designs due to a lack of statistical plans in a preexisting protocol or missing the blinding process. These standards are high, however, for behavioral trials. Behavioral trials have only recently adopted standards regarding registration of protocols and data analysis plans. Such standards have historically been ‘optional’ for behavioral trials while regulatory organizations (e.g., the Food and Drug Administration) have required them for medication trials. Similarly, blinding is a real challenge for behavioral trials. It is impossible to blind a person to treatment when that treatment requires active engagement in physical exercise, cognitive training, psychotherapy, or the like. Rather, behavioral trials must attempt to be contended with expectancy (aka placebo) and practice effects by using active control groups and/or contact-time controls as was done in a few of the trials described above. Our preference for ‘untreated’ controls in systematic reviews and meta-analyses may therefore invite higher estimates of bias in behavioral studies. All the studies included cognition as an outcome variable while seven studies reported findings on mood. Results indicated low heterogeneity in cognition even after nesting outcomes within studies and in mood. Funnel plots and the adjusted Egger’s test both supported the lack of publication bias in both outcomes. However, since there were fewer than 10 reports for mood, the results might not obtain sufficient power.

**Figure 6:**
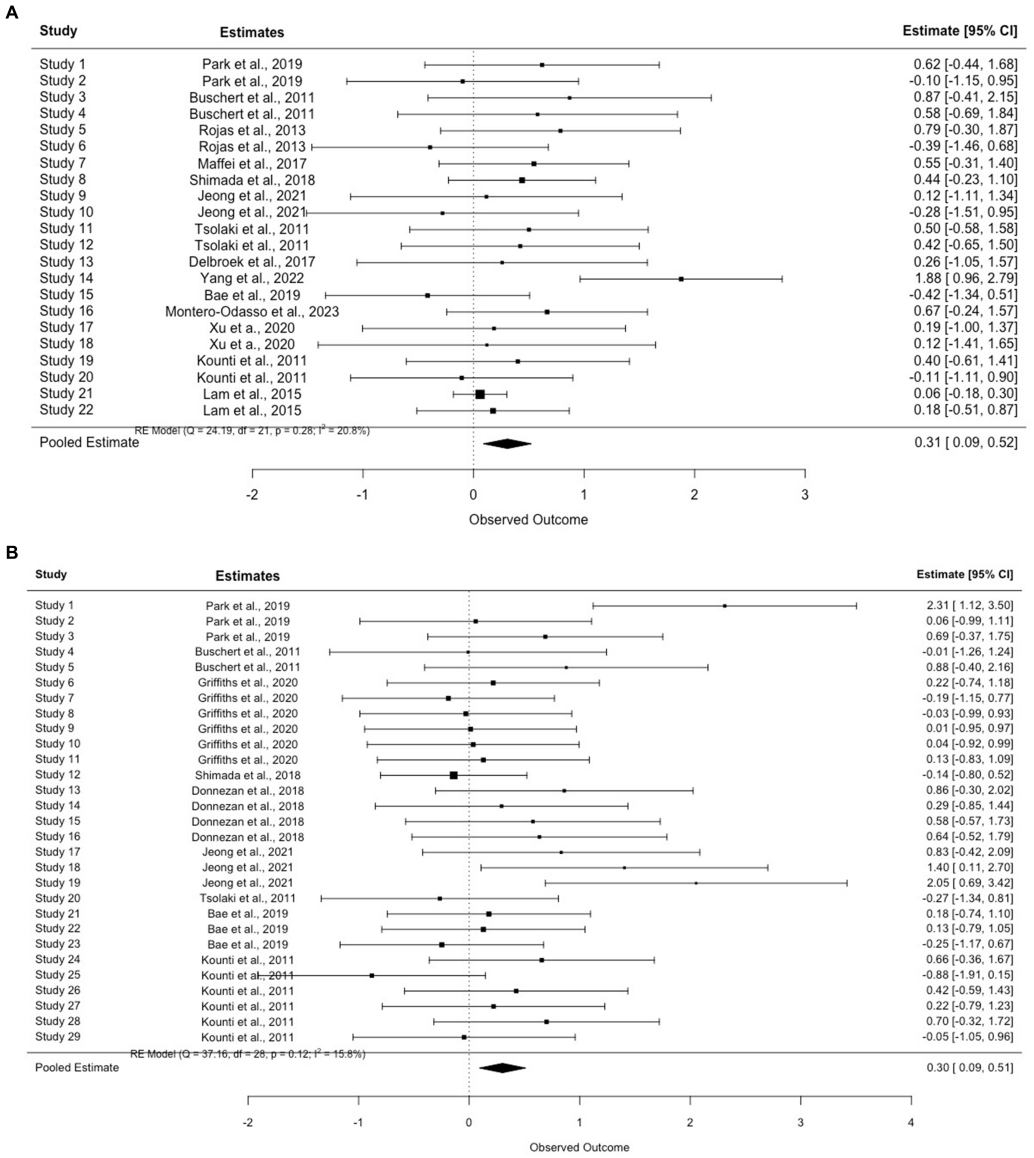

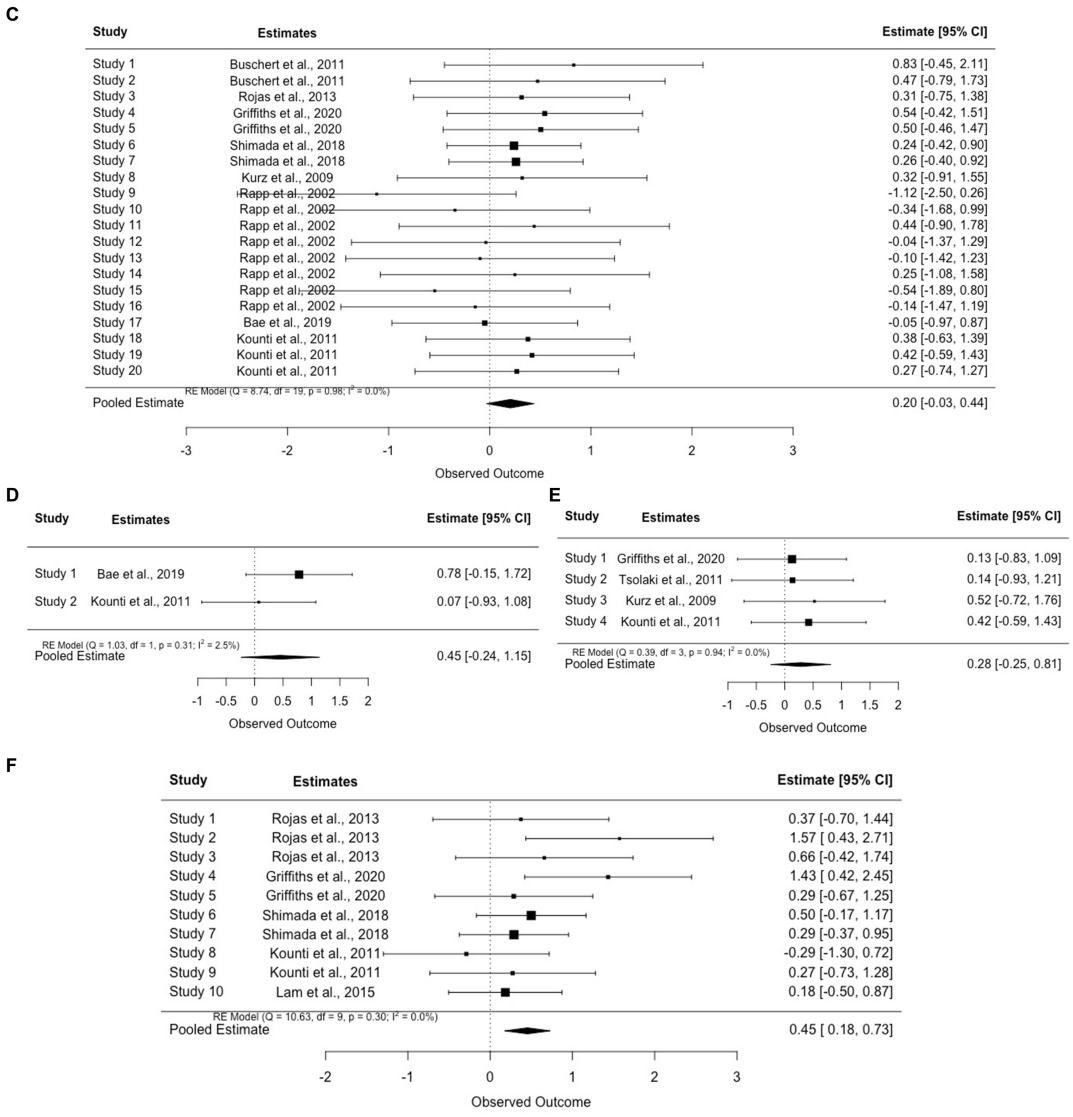
**(A)** Forest plot for global cognition. **(B)** Forest plot for executive function. **(C)** Forest plot for verbal memory. **(D)** Forest plot for non-verbal memory. **(E)** Forest plot for visuospatial ability. **(F)** Forest plot for semantic fluency.

Overall, multimodal cognitive and behavioral interventions for pwMCI had a small to medium effect (*k* = 18, *g* = 0.41, 95% CI = [0.21–0.67]) on cognition. Due to the complexity and diversity of cognitive outcomes, effect sizes were aggregated from available cognitive scores. Therefore, a *post hoc* analysis of focused cognitive domains was conducted. Specifically, global cognition improved in most of the studies (*k* = 14) except for Delbroek et al. (2017), Xu et al. (2020), and Bae et al. (2019). A subgroup meta-analysis demonstrated a small-moderate effect on global cognition (*k* = 14, *g* = 0.31, 95% CI = [0.09, 0.52]) (Figure 6A). However, benefits observed by the end of treatment might not be preserved in the long term. In the follow-up study Buschert et al. (2012) noted that the significant main effect of MMSE (*F*(1,18) = 8.50, *p* < 0.01,η^2^ = 0.23) observed in Buschert et al. (2011) mitigated at 15-month and 28-month (*F*(1,16) = 4.91, *p* = 0.041, η^2^ = 0.23) while ADAS-cog stably improved (*F*(1,18) = 6.38, *p* = 0.021, η^2^ = 0.26).

Verbal (*k* = 8) and non-verbal memory (*k* = 2) were also commonly measured. Similarly, small-moderate effects were found in each domain (verbal memory (*g* = 0.20, 95% CI = [−0.03, 0.44]) and non-verbal memory (*g* = 0.45, 95% CI = [−0.24, 1.15])). See Figures 6C,D. In general, almost all the studies that included cognitive training also included memory as one of the major targeted training domains. Therefore, it was not surprising to observe improvement in verbal and nonverbal memory tests across studies with only one exception (Rojas et al., 2013). Nevertheless, instead of traditional memory training, Rojas et al. (2013) emphasized episodic memory encoding strategies via visual imagery, semantic knowledge, and executive control. This approach was commonly used to improve the speed of processing, attention, and useful memory instead of verbal memory. Aside from cognitive stimulation, cognitive training provided in this intervention involved theoretically motivated cognitive strategies to improve metacognition and self-efficacy in taking control of cognition. Thus, while the authors did not explain the lack of improvement of verbal memory, a potential reason might be related to the reduced capability to sufficiently exploit learned memory skills due to declined executive function and semantic ability.

Benefits on other cognitive domains have also been demonstrated repeatedly across studies (e.g., executive function (*k* = 9, *g* = 0.30, 95% CI = [0.09, 0.51]) and visuospatial skills (*k* = 4, *g* = 0.28, 95% CI = [−0.25, 0.81])). See Figures 6B,E. Training using dual-task games (e.g., playing memory games while pedaling) revealed significant improvements in executive function including speed of processing, reasoning, and inhibition. For example, Jeong et al. (2021) asked participants to complete cognitive tasks such as speaking and counting while doing fifty-minute of aerobic exercises and found improvement in processing speed, particularly in fast switching between letters and numbers (TMT-B; *p* < 0.01) or matching symbols to numbers according to a key (Digit Symbol Substitution Test; *p* < 0.01). The authors attributed this improvement to increased regular physical exercises and argued that changes in executive function were important for dementia prevention because both executive function and attention were significant predictors of AD in pwMCI (Jacobs et al., 1995).

Verbal fluency measured through semantic and category fluency tests was the domain with the lowest pooled effect size compared to other domains (*g* = 0.45, 95% CI = [0.18, 0.73]) (Figure 6F). Among the studies that assessed changes in verbal fluency and confrontational naming, two (Shimada et al., 2018; Griffiths et al., 2020) reported significant improvements while one noted comparable changes in both groups (Rojas et al., 2013; control: mean change = 2.40, *p* < 0.01; intervention: mean change = 2.40, *p* < 0.01). Similar to executive function, lower verbal fluency scores in older adults with MCI could predict progression to AD. Thus, while only a few studies investigated the interaction between group and time (Kounti et al., 2011; Lam et al., 2015; Shimada et al., 2018), the superior beneficial effects supported the importance of multimodal intervention in delaying AD progression. However, a longitudinal follow-up is still warranted in these domains.

The pooled effect sizes of mood were medium to large (*k* = 7, *g* = 0.65, 95% CI = [0.37–0.93]). Two studies found no significant improvements in depressive symptoms (Bae et al., 2019; Xu et al., 2020). Notably, the improvement in mood observed in Buschert et al. (2011) was not seen at either the 15- or 28-month follow-up (Buschert et al., 2012). While multiple potential explanations were postulated by the authors, social engagement in the controls seemed to play an essential role in the studies that failed to demonstrate changes in depressive symptoms. For example, after providing group-based health education classes to the control group, Bae et al. (2019) found no between group differences in mood at the end of the intervention, which might be related to increased social engagement in both groups. Yang et al. (2022) also mentioned the comforting and supportive environment group-based interventions have provided to the patients, which might also benefit their mood symptoms. Aside from social connections, using elements of psychotherapy also appeared to improve mood in pwMCI. For instance, Kurz et al. (2009) offered extensive psychotherapy training including self-assertiveness and stress management and found a 50% reduction of depressive symptoms in the intervention group with a large effect size (*g* = 0.98). Another factor that might assist in explaining the variable results in mood was concentration difficulties. Items regarding concentration and activity level were commonly presented in depression scales, which could in turn be affected by existing cognitive deficits. Thus, Buschert et al. (2011) removed these items from their analysis and indicated that an improvement in depression might also improve the speed of processing or sustained attention. While depression improvement was not clinically significant in several reports, studies suggested that it might reflect enhancement of self-esteem and well-being, which can further benefit cognitive performance (Buschert et al., 2011).

### 4.1 Clinical implications

In the past decade, clinical trials on pharmacological interventions have not demonstrated improvement in cognition for pwMCI (Ströhle et al., 2015; Fink et al., 2018). While the FDA has recently approved Aducanumab for early stages of AD, findings did not support cognitive benefits in pwMCI (Knopman et al., 2021). Even in RCTs that showed cognitive improvement of donepezil (SMD = -0.90), the benefit was rather subtle (1 point between group difference on the 89-item ADAS-cog scale) (Doody et al., 2009). In addition, research has emphasized the frequent treatment-emergent adverse events such as diarrhea, nausea, abnormal dreams, and even increased mortality in the treatment group (Winblad et al., 2008; Doody et al., 2009). A meta-analysis of 41 RCTs has suggested small to moderate effect sizes of cholinesterase inhibitors on cognitive function (SMD = 0.10–0.46) (Cooper et al., 2013). Thus, results from this meta-analysis showed generally comparable or larger effects of multimodal nonpharmacological interventions on cognition and mood, which are consistent with previous reports (Sherman et al., 2017) and further supported the utility of these interventions to maintain functionality and facilitate adjustment to cognitive changes.

### 4.2 Limitations of the studies

Studies failed to mention the race and ethnicity of participants, mainly due to the homogeneity of the populations. Impacts of racial/ethnic background on the effects of multimodal or single-modal interventions have not yet been studied. Another limitation of the studies pertains to the absence of control of repeated measure effects except for Kurz et al. (2009). Because most interventions were conducted within a short time frame, a repeated testing effect at the end of the intervention, especially in cognitive tasks, might have mediated the observed changes post-intervention (Roediger and Payne, 1982). Furthermore, only one report included dementia conversion rate as an outcome (Rojas et al., 2013). Conversion to dementia was seen in one trained and three non-trained patients at the 12-month follow-up, and significant declines in global cognition were seen in the non-trained group at the six-month follow-up assessment (Rojas et al., 2013). However, since the conversion rate was low in both groups and no significant improvement was observed in the intervention group immediately after the intervention, the results need further examination to determine whether long-term effects were present. Thus, a longitudinal analysis of whether these multimodal interventions have delayed dementia progression is needed.

The Yang et al. (2022) study was found to be an outlier on the Funnel plot, indicating potential heterogeneity/publication bias. Findings in the study suggested significant cognitive improvement in the intervention group but a decline in untreated controls. Despite observed deviations from other studies, further evaluation of study population, methodology, interventions, and outcomes did not demonstrate evidence of bias or poor data quality. Therefore, we speculated that the distinctive results might stem from the relatively intense schedule for a long intervention period (6 months). The study was also unique in its short and frequent follow-ups (1-, 3-, and 6-month follow-ups). However, these hypotheses might not completely explain the reason for the deviation, and the results of Yang et al. (2022) should be interpreted with caution.

### 4.3 Limitations of the review

One of the limitations of this review is the lack of consensus in MCI diagnostic criteria across reports. Most studies included older adults with an MCI diagnosis regardless of subtype. However, four reports included only single or multidomain aMCI (Buschert et al., 2011; Park et al., 2019; Jeong et al., 2021) and one used the term mNCD and MCI interchangeably (Griffiths et al., 2020). Additionally, this study did not investigate the effects of different modes of delivery (simultaneous vs. sequential). Sequential designs were defined as delivering intervention modalities in separate sessions during the same period (e.g., exercise followed by cognitive training). In contrast, simultaneous designs were usually delivered by asking participants to perform certain cognitive tasks while exercising at the same time or by using exergaming. Most of the interventions in the current review delivered different modalities through a sequential design whereas several dual-task trainings were administered using exergaming (Delbroek et al., 2017; Donnezan et al., 2018; Shimada et al., 2018; Park et al., 2019). In healthy and cognitively impaired older adults, simultaneous training was found to be more efficacious for cognition than sequential combinations of physical exercises and cognitive training (*g* = 0.32–0.38) (Zhu et al., 2016; Gheysen et al., 2018; Gavelin et al., 2021). However, whether simultaneous or sequential delivery is superior in pwMCI has yet to be studied. An analysis to compare the modes of delivery was beyond the scope of this review. Future research could focus on differences in efficacy associated with modes of delivery.

Another limitation pertains to the number of databases searched in the study. We only searched three major databases. However, research shows that using Embase combined with PubMed can cover approximately 88% of the available literature (Frandsen et al., 2021). Previous studies have also indicated high coverage rates when combining the Cochrane Library and EMBASE (88% in hypertension systematic review) (Rathbone et al., 2016) or the three search engines (97% in orthopedic research) (Slobogean et al., 2009). Additional bibliographic databases did not provide unique records when two or three of the above databases were searched due to significant overlaps across databases (Royle and Milne, 2003; Hirt et al., 2021). The Cochrane Library was also found to have the highest precision rate in literature reviews and to be sensitive in identifying RCTs (Royle and Milne, 2003). Therefore, a combination of these three databases and a manual reference search were considered sufficient to identify all the studies meeting our inclusion criteria.

### 4.4 Conclusion and future research

Studies of multimodal cognitive and behavioral interventions on pwMCI demonstrated small to moderate positive effects on cognition and mood. A few directions for future research are postulated: (1) including long-term follow-ups to evaluate adherence and efficacy in delaying dementia conversion, (2) comparing effects of similar interventions in patients from diverse racial/ethnic backgrounds to inform adjustment in designs, and (3) considering simultaneous vs. sequential modes of delivery.

## Data availability statement

The original contributions presented in the study are included in the article/Supplementary material, further inquiries can be directed to the corresponding author.

## Author contributions

GY: Conceptualization, Data curation, Formal analysis, Investigation, Methodology, Project administration, Resources, Software, Validation, Visualization, Writing – original draft, Writing – review & editing. AP-L: Methodology, Resources, Validation, Writing – review & editing. MM: Data curation, Formal analysis, Methodology, Supervision, Writing – review & editing. S-AL: Supervision, Writing – review & editing. GS: Conceptualization, Funding acquisition, Methodology, Resources, Supervision, Validation, Writing – review & editing.
