# Supplementary material for: Multimodal cognitive and behavioral interventions for patients with MCI: a systematic review and meta-analysis on cognition and mood
Source: Front Aging Neurosci. 2024 Apr 30;16:1390699. doi: 10.3389/fnagi.2024.1390699 (PMC11091294; doi:10.3389/fnagi.2024.1390699)
Supplement: Supplementary file 1 [file Data_Sheet_1.docx]

**Supplement A. Systematic Literature review and Meta-Analysis on Multicomponent Interventions.**

| Review | K | Population | Interventions | | Outcomes | Effect Sizes |
| --- | --- | --- | --- | --- | --- | --- |
| Literature review and meta-analysis | | | | | | |
| Chandler et al., 2016 | 26 | MCI | Cognitive interventions | Mood | | d=.16 |
|  | 31 | MCI |  | Functional ability | | d=.23 |
|  | 24 | MCI |  | metacognition | | d=.30 |
| Sherman et al., 2017 | 16 | MCI | Multicomponent (i.e., different cognitive strategies) cognitive training | cognition | | g=.40 |
|  | 13 | MCI | Multi-domain (i.e., trainings targeting different cognitive domains) cognitive training | cognition | | g=.23 |
| Gavelin et al., 2021 | 32 | MCI/healthy | Cognitive training and physical exercise | Overall cognition | | g=.22 |
|  | 41 | MCI/healthy |  | Physical function | | g=.25 |
| Gheysen et al., 2018 | 29 | MCI/healthy | Physical and cognitive exercise vs control | cognition | | g=.32 |
|  | 9 | MCI |  | cognition | | g=.39 |
| Zhu et al., 2016 | 13 | Healthy | Physical and cognitive trainings vs. control | Cognition | | SMD=.29 |
| Karssemeijer et al., 2017 | 10 | MCI/dementia | Cognitive and physical interventions | global cognitive function | | SMD=.32 |
|  | 5 | MCI |  | global cognitive function | | SMD=.39 |
|  | 7 | dementia |  | global cognitive function | | SMD=.36 |
|  | 4 | MCI/dementia |  | ADL | | SMD=.65 |
|  | 4 | MCI/dementia |  | mood | | SMD=.27 |
| Yan et al., 2022 | 7 | MCI | Virtual reality combining cognitive and physical interventions | Cognition | | MD=2.66 |
| Li et al., 2022 | 9 | MCI/healthy | Taichi and cognitive training | Cognition | | SMD=.74 |
| Kiper et al., 2022 | 9 | MCI | Physical and cognitive training | Mobility | | MD=-1.80 |
| Han et al., 2022 | 17 | MCI/healthy | Physical and cognitive interventions | cognition | | N/A |
|  | 3 | MCI |  | Global cognition | | SMD = 1.40 |
|  | 2 | MCI |  | memory | | SMD = 0.70 |
|  | 1 | MCI |  | attention | | SMD = −0.04 |
|  | 2 | MCI |  | Executive function | | SMD = 0.39 |
|  | 3 | MCI |  | Depression | | SMD=.99 |
| Meng et al., 2021 | 8 | MCI | Physical and cognitive interventions vs. control | Global cognition | | SMD=.27 |
|  | 5 | MCI |  | Memory | | SMD=.29 |
|  | 7 | MCI |  | Executive function | | SMD=.23 |
| Bruderer-Hofstetter et al., 2018 | 8 | SCD/MCI | Physical exercise and cognitive training | IADLs | | SUCRA=.84 |
|  | 10 | SCD/MCI |  | Global cognition | | SUCRA=.90 |
|  | 6 | SCD/MCI |  | Executive function | | SUCRA=.81 |
|  | 10 | SCD/MCI |  | Learning and memory | | SUCRA=.89 |
|  | 9 | SCD/MCI |  | Language | | SUCRA=.77 |
|  | 7 | SCD/MCI |  | Complex attention | | SUCRA=.83 |
| Santos Lopes da Silva et al., 2023 | 6 | Healthy | Multicomponent physical trainings | Global cognition | | SMD=.58 |
|  | 4 | Healthy |  | TMT-A | | MD=-6.70 |
|  | 3 | Healthy |  | TMA-B | | MD=-8.80 |
| Literature review | | | | | | |
| Mai et al., 2022 | 7 | MCI | physical and cognitive training | | Falls | |
| Yang et al., 2020 | 10 | MCI | Cognitive and physical interventions | | Cognitive and physical outcomes | |
| *Note.* MCI=mild cognitive impairment; SCD=subjective cognitive decline; SUCRA= surface under the cumulative ranking curve, which is a Bayesian estimate of the proportion of treatment outcome in the comparison group that is worse than the one from the treatment of interest; SMD=standard mean difference; MD=mean difference. | | | | | | |

**Supplement B. Searching Terms.**

***Search strategy for Embase***

Embase allows manual entry of each PICO criterion. Upon entering the targeted term, controlled vocabularies with variations (e.g., “behavior” vs. “behaviour”) and synonyms (e.g., “cognition” vs. “cognitive functioning”) showed up for selection. After accommodating to the controlled and candidate words and applicable synonyms, the following terms were entered into Embase search bar on 08/14/2023, which yielded 90 results.

*Search:* ('mild cognitive impairment'/exp OR 'amnestic mild cognitive impairment' OR 'mild cognitive impairment') AND (('multimodal intervention'/exp OR 'multi target' OR ‘multicomponent’) AND 'behavioural intervention' OR 'behavioral intervention' OR 'behavioral treatment' OR 'behavioural treatment' OR 'behavioral training' OR 'behavioural training' OR 'cognitive training'/exp OR 'cognitive intervention' OR 'cognitive treatment') AND ('controlled clinical trial'/exp OR 'clinical trial, controlled' OR 'controlled clinical comparison' OR 'controlled clinical drug trial' OR 'controlled clinical experiment' OR 'controlled clinical study' OR 'controlled clinical test' OR 'controlled clinical trial' OR 'treatment'/exp) AND ('cognition'/exp OR 'cognition' OR 'cognitive function' OR 'cognitive symptoms' OR 'daily life activity'/exp OR 'adl (activities of daily living)' OR 'activities of daily living' OR 'activity, daily living' OR 'daily life activity' OR 'daily living activity' OR 'mood'/exp OR 'mood' OR 'psychologic mood' OR 'memory'/exp OR 'item recall' OR 'memory' OR 'memory function' OR 'nonspatial memory' OR 'remembering' OR 'reminiscence' OR 'anxiety'/exp OR 'anxiety' OR 'depression'/exp OR 'clinical depression' OR 'depression' OR 'depressive disease' OR 'depressive disorder' OR 'depressive episode' OR 'depressive illness' OR 'depressive state' OR 'depressive symptom' OR 'depressive syndrome' OR 'mental depression') AND ('randomized controlled trial'/exp OR 'controlled trial, randomized' OR 'randomised controlled study' OR 'randomised controlled trial' OR 'randomized controlled study' OR 'randomized controlled trial' OR 'trial, randomized controlled')

***Search strategy for PubMed***

The following terms were searched on PubMed on 08/09/2022, which yielded 104 results. Search was limited to randomized controlled trials. Among the results, 8 were removed due to duplication.

*Search:* (mild cognitive impairment or (mci or amci or mci-a or predement* or pre-dement* or pre-AD or (mild cogniti* (decline or impair* or deficit* or deteriorate* or disorder*)) or ((prelude or preclinical or pre-clinical or prodromal or precursor) (dement* or Alzheimer* or AD))) AND (cognitive therapy or ((cogniti* or behavi* or memory or attention or information or neuropsychological or rehearsal* or mnemonic) (intervention* or rehab* or program* or strategy* or train* or retrain* or treatment* or therapy or therapies or stimulat* or technique*)) AND (("multicomponent"[All Fields]) OR ("multitarget"[All Fields]) OR ("multimodal"[All Fields])) AND ((treatment outcome or "quality of life" or "activities of daily living" or "mental recall" or (outcome* or efficac* or effectiv* or benefit* or ( (daily activit*) or (self* care*))) OR ("cognit*"[All Fields]) OR ("memory"[All Fields]) OR ("anxiety"[All Fields]) OR ("depression"[All Fields]))))

***Search strategy for Cochrane Library***

Cochrane Library Database manager required manual entry of individual search items or medical terms, which were then connected through AND/OR/NOT statements. Similar search from (Chandler et al., 2016) was entered along with the restriction of multimodal studies. Search was limited to trials and Cochrane reviews up to July 2022. This search yielded 208 results, among which 204 were clinical trials and 4 were Cochrane reviews. Among all the reports generated in this search, 29 duplicated Embase and 62 duplicated PubMed.

*Search:*

#1 mild cognitive impairment OR (mci OR amci OR mci-a OR predement* OR pre-dement* OR pre-AD OR (mild cogniti* (decline or impair* or deficit* or deteriorate* or disorder*)) or ((prelude or preclinical or pre-clinical or prodromal or precursor) AND (dement* or Alzheimer* or AD)))

#2 cognitive therapy OR (cogniti* OR behavi* OR memory OR attention OR information OR neuropsychological OR rehearsal* OR mnemonic) (intervention* or rehab* or program* or strategy* or train* or retrain* or treatment* or therapy or therapies or stimulat* or technique)

#3 #1 AND #2 in Trials

#4 multimodal OR multicomponent OR multitarget OR multifaceted

#5 #3 AND #4

#6 limit #5 to (addresses or autobiography or bibliography or biography or case reports or classical article or comment or dictionary or directory or editorial or historical article or in vitro or interactive tutorial or interview or legal cases or legislation or letter or news or newspaper article or patient education handout or periodical index or portraits or technical report)

#7 #5 NOT #6

#8 (infant* OR infancy OR newborn* OR baby* OR babies OR neonat* OR preterm* OR prematur* OR postmatur* OR child* OR schoolchild* OR school age* OR preschool* OR kid or kids OR toddler* OR adoles* OR teen* OR boy* OR girl* OR minors* OR pubert* OR pubescen* OR prepubescen* OR paediatric* OR paediatric* OR peadiatric* OR nursery school* OR kindergar* OR primary school* OR secondary school* OR elementary school* OR high school* OR highschool* or youth)

#9 #7 NOT #8

#10 #9 NOT (exp neoplasms or epilepsy or (schizophren* or cancer* or neoplas* or epilep*))

#11 (treatment outcome or treatment failure or activities of daily living or mental recall or (outcome* or efficac* or effectiv* or benefit* or ((daily activit*) or (self* adj care*1))) or ((pre* or post*) adj interven*))

#12 #10 AND #11

**Supplement C. List of studies excluded after full-text review**.

Damirchi, A., Hosseini, F., & Babaei, P. (2018). Mental training enhances cognitive function and BDNF more than either physical or combined training in elderly women with MCI: a small-scale study. *American Journal of Alzheimer’s Disease & Other Dementias®*, *33*(1), 20–29.

Diamond, K., Mowszowski, L., Cockayne, N., Norrie, L., Paradise, M., Hermens, D. F., Lewis, S. J. G., Hickie, I. B., & Naismith, S. L. (2015). Randomized controlled trial of a healthy brain ageing cognitive training program: effects on memory, mood, and sleep. *Advances in Alzheimer’s Disease*, *4*, 355‐365. https://doi.org/10.3233/978-1-61499-542-5-355

Forster, S., Buschert, V. C., Teipel, S. J., Friese, U., Buchholz, H.-G., Drzezga, A., Hampel, H., Bartenstein, P., & Buerger, K. (2011). Effects of a 6-month cognitive intervention on brain metabolism in patients with amnestic MCI and mild Alzheimers Disease. *Journal of Alzheimer’s Disease*, *26*(SUPPL. 3), 337‐348. https://doi.org/10.3233/JAD-2011-0025

Hagovská, M., & Olekszyová, Z. (2016). Impact of the combination of cognitive and balance training on gait, fear and risk of falling and quality of life in seniors with mild cognitive impairment. *Geriatrics and Gerontology International*, *16*(9), 1043–1050. https://doi.org/10.1111/ggi.12593

Han, J. W., Lee, H., Hong, J. W., Kim, K., Kim, T., Byun, H. J., Ko, J. W., Youn, J. C., Ryu, S. H., Lee, N. J., & al., et. (2017). Multimodal Cognitive Enhancement Therapy for Patients with Mild Cognitive Impairment and Mild Dementia: a Multi- Center, Randomized, Controlled, Double-Blind, Crossover Trial. *Journal of Alzheimer’s Disease*, *55*(2), 787‐796. https://doi.org/10.3233/JAD-160619

Jeong, J. H., Na, H. R., Choi, S. H., Kim, J., Na, D. L., Seo, S. W., Chin, J., Park, S. A., Kim, E.-J., Han, H. J., Han, S.-H., Yoon, S. J., Lee, J.-H., Park, K. W., Moon, S. Y., Park, M. H., Choi, M. S., Han, .I-W., Lee, J. H., … Kim, J. Y. (2016). Group-and home-based cognitive intervention for patients with mild cognitive impairment: A randomized controlled trial. *Psychotherapy and Psychosomatics*, *85*(4), 198–207. https://doi.org/10.1159/000442261

Kim, K. W., Han, J. W., Yoon, J. C., Ryu, S.-H., Lee, N.-J., Hong, J. W., Kim, K. Y., & Kim, T. H. (2015). Effects of multimodal cognitive enhancement therapy (MCET) for people with mild cognitive impairment and early stage dementia: a randomized, controlled, double-blind, cross-over trial. *Alzheimer’s & Dementia*, *11*(7 SUPPL. 1), P465. https://www.cochranelibrary.com/central/doi/10.1002/central/CN-01163333/full

Kinsella, G., Ames, D., Storey, E., Ong, B., Pike, K., Mullaly, E., Rand, E., Clare, L., Saling, M., & Parsons, S. (2012). Knowledge-transfer following cognitive intervention for amnestic mild cognitive impairment. *Alzheimer’s and Dementia*, *8*(4), P235. https://doi.org/10.1016/j.jalz.2012.05.622

Montero-Odasso, M., Almeida Quincy, J., Burhan Amer, M., Camicioli, R., Doyon, J., Fraser, S., Li, K., Liu-Ambrose, T., Middleton, L., Muir-Hunter, S., & al., et. (2018). SYNERGIC TRIAL (SYNchronizing Exercises, Remedies in Gait and Cognition) a multi-Centre randomized controlled double blind trial to improve gait and cognition in mild cognitive impairment. *BMC Geriatrics*. https://doi.org/10.1186/s12877-018-0782-7

Naismith, S. L., Pye, J., Terpening, Z., Lewis, S., & Bartlett, D. (2019). “Sleep Well, Think Well” Group Program for Mild Cognitive Impairment: a Randomized Controlled Pilot Study. *Behavioral Sleep Medicine*, *17*(6), 778‐789. https://doi.org/10.1080/15402002.2018.1518223

Singh, M. A. F., Gates, N., Saigal, N., Wilson, G. C., Meiklejohn, J., Brodaty, H., Wen, W., Singh, N., Baune, B. T., & Suo, C. (2014). The Study of Mental and Resistance Training (SMART) study—resistance training and/or cognitive training in mild cognitive impairment: a randomized, double-blind, double-sham controlled trial. *Journal of the American Medical Directors Association*, *15*(12), 873–880.

Styliadis, C., Kartsidis, P., Paraskevopoulos, E., Ioannides, A. A., & Bamidis, P. D. (2015). Neuroplastic effects of combined computerized physical and cognitive training in elderly individuals at risk for dementia: an eLORETA controlled study on resting states. *Neural Plasticity*.

Suo, C., Singh, M. F., Gates, N., Wen, W., Sachdev, P., Brodaty, H., Saigal, N., Wilson, G. C., Meiklejohn, J., & Singh, N. (2016b). Therapeutically relevant structural and functional mechanisms triggered by physical and cognitive exercise. *Molecular Psychiatry*, *21*(11), 1633–1642.

Tonga, J. B., Šaltytė Benth, J., Arnevik, E. A., Werheid, K., Korsnes, M. S., & Ulstein, I. D. (2021). Managing depressive symptoms in people with mild cognitive impairment and mild dementia with a multicomponent psychotherapy intervention: a randomized controlled trial. *International Psychogeriatrics*, *33*(3), 217‐231. https://doi.org/10.1017/S1041610220000216
